# Exploring the prevalence and variance of cognitive impairment, pain, neuropsychiatric symptoms and ADL dependency among persons living in nursing homes; a cross-sectional study

**DOI:** 10.1186/s12877-016-0328-9

**Published:** 2016-08-22

**Authors:** Sabine Björk, Christina Juthberg, Marie Lindkvist, Anders Wimo, Per-Olof Sandman, Bengt Winblad, David Edvardsson

**Affiliations:** 1 Department of Nursing, Umeå University, Umeå, Sweden; 2 Department of Statistics, Umeå School of Business and Economics, Umeå University, Umeå, Sweden; 3 Department of Public Health and Clinical Medicine, Epidemiology and Global Health, Umeå University, Umeå, Sweden; 4 Department of Neurobiology, Care Sciences and Society, Division of Neurogeriatrics, Karolinska Institutet, Stockholm, Sweden; 5 Department of Neurobiology, Care Sciences and Society, Division of Nursing, Karolinska Institutet, Stockholm, Sweden; 6 Department of Health Sciences, Luleå University of Technology, Luleå, Sweden; 7 School of Nursing and Midwifery, La Trobe University, Melbourne, Australia

**Keywords:** Nursing homes, Frail elderly, Dementia, Pain, Activities of daily living, Neuropsychiatric symptoms, Prevalence, Cross-sectional study

## Abstract

**Background:**

Earlier studies in nursing homes show a high prevalence of cognitive impairment, dependency in activities of daily living (ADL), pain, and neuropsychiatric symptoms among residents. The aim of this study was to explore the prevalence of the above among residents in a nationally representative sample of Swedish nursing homes, and to investigate whether pain and neuropsychiatric symptoms differ in relation to gender, cognitive function, ADL-capacity, type of nursing-home unit and length of stay.

**Methods:**

Cross-sectional data from 188 randomly selected nursing homes were collected. A total of 4831 residents were assessed for cognitive and ADL function, pain and neuropsychiatric symptoms. Data were analysed using descriptive statistics and the chi-square test.

**Results:**

The results show the following: the prevalence of cognitive impairment was 67 %, 56 % of residents were ADL-dependent, 48 % exhibited pain and 92 % exhibited neuropsychiatric symptoms. The prevalence of pain did not differ significantly between male and female residents, but pain was more prevalent among cognitively impaired and ADL-dependent residents. Pain prevalence was not significantly different between residents in special care units for people with dementia (SCU) and general units, or between shorter-and longer-stay residents. Furthermore, the prevalence of neuropsychiatric symptoms did not differ significantly between male and female residents, between ADL capacities or in relation to length of stay. However, residents with cognitive impairment and residents in SCUs had a significantly higher prevalence of neuropsychiatric symptoms than residents without cognitive impairment and residents in general units.

**Conclusions:**

The prevalence rates ascertained in this study could contribute to a greater understanding of the needs of nursing-home residents, and may provide nursing home staff and managers with trustworthy assessment scales and benchmark values for further quality assessment purposes, clinical development work and initiating future nursing assessments.

## Background

The provision of nursing-home care is a public service in Sweden, and nursing homes are individual, means-tested accommodation units provided by the municipalities for people who need full-time care. To be eligible for admission to a nursing home, the person needs extensive help in managing daily living due to age, illness and/or disability. Cognitive and physical impairments are two of the factors most associated with nursing-home admission [1] and both cognitive and ADL impairments have been shown to be common in nursing homes internationally [2–5]. Two previous studies in Sweden show that cognitive impairment among nursing-home residents was prevalent in 71 % (2008) [6] and 73 % (2013) [7] respectively of all cases. However, these studies were limited because of their regional context and, in the case of the first one, also regarding their sample size (*n* = 315). Studies in similar settings from other countries indicate rates ranging from 59 % (2009) [2] to 68 % [3] and as high as 80 % (2004/2005) [4]. Among US nursing-home residents, only 11.0 % (2012) were reported to have little or no cognitive impairment and no ADL impairment [5]. In a European study (2009–2011) in eight countries (the Czech Republic, England, Finland, France, Germany, Italy, The Netherlands and Israel) including approximately 500 residents from each country, 81 % of nursing-home residents are described as needing assistance or dependent in ADL. The study used the seven point MDS Activities of Daily Living Hierarchy scale [8] to measure ADL, where a score of 2–4 indicates ADL assistance, and a score of ≥ 5 being dependent in ADL [3]. Although the study by Onder et al. had a large sample it was not randomized.

Previous studies also show pain to be a prevalent symptom among older people in nursing homes and similar settings [9–12]. A Swedish cross-sectional study (2000) reported a pain prevalence of 57 % among residents in nursing homes [11]. Results from the European study (2009–2011) of eight countries (excluding Sweden) show a prevalence of pain ranging from 20 % (Israel) to 78 % (Finland) [12] and a review of 27 studies (1990 to 2009) finds pain to be common among residents but the prevalence varied from 4 to 80 % among the different studies. The highest rates were found when pain was self-reported, around 60–70 %, with a range of 28 to 80 %. The prevalence of pain varied from 4 to 64 % when information was collected by means of chart review or data from the MDS database. When mixed methods were used, such as interview together with observation, the prevalence rates were around 40–60 % [9]. Differences in the prevalence of pain and its association with gender, cognitive impairment, ADL capacity and type of unit have also been described. The prevalence of pain was found to be higher among female residents [13, 14]. Pain has been positively associated with female gender and negatively associated with dementia and severe cognitive impairment [10, 15]. No differences in prevalence were seen between residents with and without cognitive impairment in the Swedish study [11]. Additionally, in a US study with a sample of 13 107 nursing-home residents, residents in special care units for people with dementia (SCU) were found to have less pain than residents in regular units [16].

Exhibiting neuropsychiatric symptoms is another leading cause for admission to a nursing home [17]. Neuropsychiatric symptoms have a multifactorial aetiology which includes disease-related neuropathological changes, unmet physical or psychological needs, environmental influence and/or pain [18]. A review of studies (1987 to 2012) from Spain, The Netherlands, Finland, Austria, the UK, USA, Korea, Japan Taiwan, and Australia indicates that the prevalence of one or more neuropsychiatric symptoms ranged from 38–95 % in nursing-home residents with dementia [19]. A Dutch study (2011) shows a prevalence of 89 % among residents in SCUs [20], and male residents with dementia are described as exhibiting more neuropsychiatric symptoms [21, 22]. Neuropsychiatric symptoms are also described as being associated with impaired ADL function [23].

As illustrated in the above studies, the prevalence of cognitive impairment, pain and neuropsychiatric symptoms varies among countries and contexts, and prevalence rates also seem to vary among resident characteristics such as gender, cognitive impairment, and ADL impairment, as well as type of unit. The prevalence of pain seems to be higher among female residents and lower among residents with cognitive impairment, even though conflicting results exist [11]. This indicates a need to further study the prevalence and variance in prevalence rates on national levels. There seems to be a shortage of national randomized population-based studies on the prevalence of these symptoms. Cross-sectional data have been collected from nursing-home residents in Nordic countries, but limited to residents in the four capitals of Denmark, Finland, Iceland and Sweden, as well as at a European level [3, 10, 12] but since Sweden has no national repeated measures of health and health-related factors for the national population of residents in nursing homes as other countries have, comparison of studies is difficult. Large [7, 11] and smaller [6, 24] studies have been performed in Swedish nursing homes, but to our knowledge no national study of the prevalence of cognitive impairment, pain and neuropsychiatric symptoms among residents in nursing homes has been reported. Knowledge about national prevalence rates would provide a point of reference that could be of great value for other future national and international studies, allowing comparisons of their result with these results. National evaluation of residents’ needs and capabilities would help in questions of financing and the planning of care on a national level, and thus also be of value to stakeholders and organizations. Knowledge about national prevalence rates could contribute to a greater understanding of nursing-home residents’ needs, and would provide nursing-home staff and researchers with the starting point for future nursing assessments.

The aim of this study was, therefore, to explore the prevalence of cognitive impairment, ADL-dependency, pain and neuropsychiatric symptoms among residents in a nationally representative Swedish sample, and to investigate whether pain and neuropsychiatric symptoms differ in relation to gender, cognitive function, ADL-capacity, type of unit and length of stay.

The following research questions were explored:

- What is the prevalence of cognitive impairment, ADL-dependency, pain and neuropsychiatric symptoms among older people in Swedish nursing homes?
- Does the prevalence of pain and neuropsychiatric symptoms differ in relation to gender, cognitive function, ADL-capacity, type of unit and length of stay?

## Methods

### Design, sampling and participants

The data for the analyses in this study were drawn from the cross-sectional baseline data set from the Swedish National Inventory of Health and Care in Nursing Homes (SVENIS) [25], longitudinal studies are planned with the next one to be conducted in 2018. Data were collected between November 2013 and September 2014. From a randomly selected sample of 60 of the 290 Swedish municipalities, 38 agreed to participate in the study. In these 38 municipalities, 194 nursing-home facilities were contacted about participation, and all but six agreed. Unit managers were contacted by telephone, given information about the study and sent questionnaires. These were then distributed to staff who conducted all assessments of residents. Only residents permanently living in the participating nursing homes were included in the study. Hence, residents subject to short-term or temporary arrangements were excluded. Staff members were provided with written information about the study and with instructions on how to carry out the assessments. No personal identification of the individual residents or staff was collected.

### Data collection

Unit managers informed the staff about the study. The member of staff who knew each individual resident best was asked to perform the proxy ratings, based on his/her professional assessment of the individual resident.

### Instrumentation

Cognitive function was assessed using the Gottfries’ cognitive scale, consisting of 27 items, intended to measure a person’s level of cognitive function [26]. Items are formulated as statements answered with a ‘yes’ (1) or ‘no’ (0), and higher scores indicate a higher cognitive function. Scores lower than 24 indicate cognitive impairment. Internal consistency was found to be satisfactory in the dataset with a Cronbach’s alpha of 0.96. Criterion-related validity of the cut-off of the scale has been established against the Mini-Mental State Examination [27].

ADL capacity was measured using a modified version of the Katz Index of Independence in Activities of Daily Living [28]. The scale assesses the ability to independently manage daily activities in the following six personal ADL domains: bathing, dressing, transferring, toileting, eating, and continence. Each domain was scored dichotomously as dependent ‘0’ versus fully independent ‵1′, giving a total score of 0–6 points. Higher scores indicate greater independence. Internal consistency was satisfactory in the dataset as evidenced by a Cronbach’s alpha of 0.86.

Pain was investigated using the Pain Assessment in Advanced Dementia (PAINAD) scale, which evaluates symptoms of pain based on breathing, body language, facial expression, vocalization, and consolability. Each of the five items is scored between 0 and 2, giving a total score of 0–10 points; higher scores indicate greater pain intensity [29]. Criterion validity has been reported as satisfactory [30] internal consistency values of 0.5–0.65 have been reported [29]. A Swedish version of the instrument was available, but no published data on validity were found. PAINAD has also been tested in hospitalized cognitively intact and impaired post-orthopaedic surgical older adults and a positive correlation was found between a self-report pain scale and the PAINAD [31]. The internal consistency was satisfactory in the dataset as evidenced by a Cronbach’s alpha of 0.75.

The prevalence of neuropsychiatric symptoms was assessed using the Neuropsychiatric Inventory—Nursing Home Version (NPI-NH). The NPI-NH evaluates the following 12 neuropsychiatric symptoms: delusions, hallucinations, agitation/aggression, depression/dysphoria, anxiety, elation/euphoria, apathy, disinhibition, irritability/lability, aberrant motor behaviours, night-time behaviours, and eating behaviours. Each symptom is rated from not occurring at all (a score of 0) to occurring several times per day (a score of 4). The NPI-NH has previously been found to be valid and reliable [32], for example in a Norwegian version with high inter-rater reliability values (inter-rater reliability between 0.85 and 1.0, across assessors with different types of health education) and high internal consistency values (Cronbach’s α >0.8) [33]. There is a Swedish version of the instrument which is available and widely used but no published data on validity were found. The internal consistency was satisfactory in the dataset as evidenced by a Cronbach’s alpha of 0.83.

### Statistics

Participant characteristics and prevalence of symptoms were explored using descriptive statistics. To test relationships between resident characteristics (sex, cognitive function, ADL-capacity, type of unit and length of stay) and the prevalence of symptoms (pain and neuropsychiatric symptoms), chi-square tests for independence with Yates’ continuity correction were used with the φ coefficient as the effect size measure. A Yates’ continuity correction was performed as chi-square tests are biased upwards in 2 × 2 contingency tables. An upwards bias tends to make the result more significant than it should be. To account for the increased risk of making type 1 errors when performing multiple tests, differences were regarded as being statistically significant when *p*-values were <0.01 (instead of the more commonly used 0.05). The φ-coefficient >0.1 was used based on criteria suggested by Cohen [34].

For the analyses, scores on the Gottfries’ cognitive scale, P-ADL, PAINAD, NPI-NH and resident length of stay were coded into dichotomous variables. The Gottfries’ cognitive scores were dichotomized in two groups where a score of 0–23 indicates a cognitive impairment equal to dementia, and a score of 24–27 indicates being cognitively intact [14, 27]. The ADL index was dichotomized into scores of 0–3 indicating ADL dependence and 4–6 indicating ADL independence [35]. The NPI-NH scores were dichotomized as ‵0′ if symptoms were not present and ‵1′ if symptoms were present. PAINAD scores were dichotomized in two groups where a score of 0 or 1 indicates no pain and a score of 2–10 indicates pain [36]. The sample median length of stay (21.5 months) was used to determine the shortest and longest length of stay. To explore the degree of cognitive impairment in the sample, the Gottfries’ cognitive scores were further divided into mild cognitive impairment (score 16–23), moderate cognitive impairment (score 8–15) and severe cognitive impairment (score 0–7) [37]. Missing data were not replaced. All statistical analyses were conducted using Statistical Package for Social Sciences version22 (for Windows; IBM, Armonk, NY, USA).

## Results

A total of 6 902 resident questionnaires were distributed, and 4 831 were completed and returned, giving an overall response rate of 70 %. The 188 participating nursing home facilities had between 7 and 128 beds and included both SCU (31 %), and general units (69 %). The staff who performed proxy assessment of the residents were mostly female (94 %) and worked as enrolled nurses (84 %). More than half (58 %) of the staff reported that they interacted with the resident on a daily, working basis and the rest reported that they interacted with resident on a weekly basis (42 %). The resident sample (*N* = 4831) contained more female (67.8 %) than male (32.2 %) residents and the resident age ranged from 47–107 years with a mean age of 85.5 years. The sample median length of stay was 21.5 months and ranged from 0–379 months. A minority of residents (17.6 %) had Swedish as a second language and 37.8 % of the residents lived in a SCU (Table 1).

**Table 1:** Sample characteristics

	% (n)	95 % CI	Missing (n)
Female	67.8 (3239)	66.5–69.1	54
Residing with partner	2.9 (135)	2.4–3.3	99
Single room	94.9 (4534)	94.3–95.6	55
Swedish as first language	82.4 (3844)	81.3–83.5	167
Residing in SCU	37.8 (1778)	36.4–39.1	122
Residing in regular unit	62.2 (2931)	60.9–63.6	
ADLs			
Showering/Bathing	81.0 (3848)	79.8–82.1	78
Dressing	68.4 (3237)	67.1–69.8	102
Toileting	53.1 (2512)	51.7–54.5	101
Transferring	43.7 (2070)	42.2–45.1	89
Continence	49.1 (2281)	47.7–50.5	185
Eating	15.8 (748)	14.8–16.9	106
Resident age, years (Mean ± SD)	85.5 (7.8)		323
Length of stay, months (Mean ± SD)	30.4 (32.0)		1439

Percentages are in valid percent, N does not add up to 4831 in all variables due to missing data

The results showed that 66.6 % of residents were rated as having a cognitive impairment and 56.3 % as being ADL dependent. In addition, 92 % of the residents were assessed as presenting neuropsychiatric symptoms. Almost half of all residents were rated as having symptoms of pain (47.9 %). Of the residents with cognitive impairment, 23.6 % were rated as having severe cognitive impairment, 38.6 % moderate impairment and 37.7 % mild impairment (Table 2). More than half (52.7 %) of the residents in general units were rated as having a cognitive impairment.

**Table 2:** Prevalence of pain, cognitive impairment and neuropsychiatric symptoms

	% (n)	95 % CI	Missing (n)
ADL dependent	56.3 (2526)	54.9–57.8	347
Pain	47.9 (2103)	46.4–49.4	441
Cognitive impairment	66.6 (2827)	65.2–68.0	586
Mild	37.7 (1067)	36.0–39.5	−
Moderate	38.6 (1092)	36.8–40.4	−
Severe	23.6 (668)	22.1–25.2	−
Neuropsychiatric symptom, any	92.0 (4309)	91.2–92.8	146

Percentages are in valid percent, N does not add up to 4831 in all variables due to missing data

The results also show that the prevalence of pain was not significantly different between male and female residents. Pain was more prevalent among residents with cognitive impairment and those who were ADL-dependent. There was a significant difference between SCUs and general units regarding the prevalence of pain but the effect size was very small, <0.1, and was interpreted as unimportant. The prevalence of pain did not differ significantly between shorter-and longer-stay residents. The prevalence of neuropsychiatric symptoms did not differ significantly between resident gender, ADL capacity or length of stay. However, significant differences were found between residents with and without cognitive impairment and between residents living at SCUs and general units (Table 3).

**Table 3:** Differences in prevalence of pain and neuropsychiatric symptoms by resident characteristics, type of unit and resident length of stay

Characteristics, % (n)	Pain	*p*	E.S.	One or more NPS	*p*	E.S.
Sex						
Male	45.5 (634)	0.038	0.032	91.8 (1371)	0.839	0.004
Female	49.0 (1448)			92.1 (2895)		
Cognitive function						
Intact	**31.0** (**407**)	<**0.001**	**0.237**	**83.8** (**1134**)	<**0.001**	**0.195**
Impaired	**56.2** (**1475**)			**95.3** (**2650**)		
ADL capacity						
Independent	**35.7** (**642**)	<**0.001**	**0.213**	88.7 (1676)	<0.001	0.096
Dependent	**57.2** (**1335**)			94.1 (2332)		
Type of unit						
General	44.4 (1191)	<0.001	0.093	**89.7** (**2547**)	<**0.001**	**0.102**
SCU	54.0 (871)			**95.4** (**1657**)		
Length of stay, median split						
Short	43.9 (693)	0.001	0.059	90.9 (1507)	0.075	0.032
Long	49.7 (785)			92.7 (1537)		

Bold text indicates statistically significant differences (*p* <0.01) with effect size (E.S) of, φ >0.1. *NPS* neuropsychiatric symptoms, Short <21.5 months, Long >21.5 months

## Discussion

This study aimed to explore the prevalence of cognitive impairment, ADL-dependency, pain and neuropsychiatric symptoms among residents in a nationally representative Swedish sample, and to investigate whether pain and neuropsychiatric symptoms differ in relation to gender, cognitive function, ADL-capacity, type of unit and length of stay. The results found in this study provide a deeper knowledge about the national prevalence rates, which could contribute to a greater understanding, and awareness of the needs of nursing-home residents. The findings presented in this study are baseline data that will enable follow-up studies. A longitudinal follow-up is planned for 2018 and other future national and international studies can compare those results with the results from this study. The results show a cognitive impairment prevalence rate of 67 %, which is in line with earlier studies in similar contexts which indicate prevalence rates of 71–73 % [6, 7]. This finding is also in line with a European study including 4156 residents in eight countries (the Czech Republic, England, Finland, France, Germany, Italy, The Netherlands and Israel) which shows an overall prevalence of cognitive impairment of 68 % [3]. Thus, the results of this study support previous studies and contribute recent data to the literature concerning cognitive impairment in nursing homes.

The results also show that about 56 % of residents were ADL-dependent, which is higher than the overall prevalence of dependency reported in a previous European study (40 %) [10]. However, resident ADL capacity was evaluated using different scales and cannot therefore be directly compared. Pain was prevalent among almost half of the residents (48 %), a finding which is congruent with other European studies showing prevalence rates of 48 % [10], but lower than a previous Swedish study reporting a prevalence rate of 57 % [11]. Again, different pain assessment methods were used in these studies which could help to explain the differences. The results of this study indicate that the prevalence of pain was higher among residents with cognitive impairment and among ADL-dependent residents, but did not differ in relation to resident gender. These findings are incongruent with previous findings from a Swedish study showing that the prevalence of pain did not differ in relation to cognitive impairment [11] and that the prevalence was higher among female residents [14]. The prevalence of pain in this study can be interpreted as indicating that further assessment and management of pain could be a priority in Swedish nursing homes.

This study also showed a high prevalence of cognitive impairment in regular units (53 %), which can be compared to slightly lower prevalence rates (40 %) reported in a recent UK study [38]. It has been suggested that dementia continues to be under-diagnosed in general nursing home units [39–41], an earlier Swedish study, for example, found that although 71 % of the sample were assessed as having a cognitive impairment, only 40 % had a dementia diagnosis [6]. The high prevalence of cognitive impairment in general units in this study may at least be partly explained by a reduction in the number of nursing-home beds in Sweden since the early 2000, and the associated higher thresholds for nursing-home admission. As a result of these changes, nursing-home residents are older and more frail when admitted to nursing homes today compared to previously [24]. It seems that to facilitate person-centred care for residents, further clinical assessments may be appropriate to determine the presence and type of dementia. Further studies would be valuable.

The results of this study also show that a majority of the cognitively impaired residents presented with one or more neuropsychiatric symptoms (95 %), which is slightly higher than the 89 % found in a previous Dutch study [20]. In addition to being a biomedical symptom, neuropsychiatric symptoms have been conceptualized as reflecting unmet needs, environmental overload and/or pain [42, 43]. From such a conceptualisation, the interpretation that neuropsychiatric symptoms may indicate a present underlying condition, such as pain, seems reasonable. Earlier studies have shown that increased pain treatment reduced neuropsychiatric symptoms [44–46]. Non-pharmacological interventions to manage neuropsychiatric symptoms are an essential part of contemporary geriatric and gerontological care in nursing homes, recommended by a wide range of national and international medical organizations and expert groups [47]. Such non-pharmacological interventions include environmental adjustments and person-centred activities tailored to meet residents’ needs and enhance quality of life. Very few studies have been found on the extent to which residents can participate in non-pharmacological interventions and activities in Swedish nursing homes [48], and to what extent participation in such interventions and activities is related to residents’ quality of life and thriving. In addition, results from a Swedish study in SCUs stresses that residents’ engagement in everyday activities was very low and that residents participating in such activities lived in more person-centred units and had a higher quality of life [48]. Further studies exploring the extent to which residents are engaged in non-pharmacological interventions and activities, and how this associates with indicators of wellbeing, would be valuable. Differences in the prevalence of neuropsychiatric symptoms have previously been described between males and females and in relation to level of cognitive impairment. In this study, the prevalence of neuropsychiatric symptoms did not differ significantly between resident genders, ADL capacity or length of stay. Significant differences were found between SCU and regular units, a finding that could be expected considering that the residents in the former have a cognitive impairment.

### Relevance for clinical practice, education and future research

Knowledge about the prevalence of symptoms and the relationships between neuropsychiatric symptoms, pain and various resident characteristics, as found in this study, could be of value in the education of students, nursing-home staff and other professions in the field. The study provides nursing-home staff and researchers with an awareness of assessment scales that could be used clinically to provide more structured evaluations, plans and targeted improvements in care. The results could also be of value as a point of reference for future national and international research studies.

### Limitations

The cross-sectional data used in this study limit the possibilities for causal inference. The findings presented are baseline data enabling the conduct of follow-up studies. Longitudinal studies are planned, the next one in 2018. Repeated longitudinal measures will allow the exploration of trends, changes over time and assessments of the impact of changes and interventions on a population level. Proxy-rating resident symptoms may also be considered a limitation of this study. However, this procedure was deliberately chosen because of the suspected, and later confirmed, high prevalence of cognitive impairment in the sample which could have compromised self-reporting. The fact that the assessors did not receive any training in assessment is a weakness of the study. However, the staff received written instructions about how to carry out the assessments. Furthermore, the assessments of residents were performed by the member of staff who knew each particular resident best, so as to increase the validity of ratings. This meant that all ratings were based on extensive personal knowledge of residents, together with the professional clinical skill of direct-care staff, safeguarding the high quality of the data. Perhaps a limitation that applies not to this study but more to comparative research overall, is the fact that different studies have used different methods to assess common symptoms. This means that differences in the prevalence of cognitive impairment, pain, and/or neuropsychiatric symptoms between this and other studies is likely to be influenced by the use of different measuring methods. Further national and international consensus statements (e.g. Moniz-Cook 2008) would be valuable [49]. There is no previously published data on validity of the Swedish language version of the PAINAD scale used in this study, which may impact the validity of the present pain ratings. Further validation estimates would be valuable.

## Conclusions

The prevalence rates ascertained in this study could contribute to a greater understanding of the needs of nursing-home residents, and may provide nursing-home staff and managers with trustworthy assessment scales and benchmark values for further quality assessment purposes, clinical development work and initiating future nursing assessments. In addition, the findings could prove helpful regarding questions regarding the financing and planning the care on a national level, and also be valuable to stakeholders and organizations.

## Abbreviations

ADL: Activities of daily living
SCU: Special care unit for people with dementia
